# Fermented Wheat Germ Alleviates Depression-like Behavior in Rats with Chronic and Unpredictable Mild Stress

**DOI:** 10.3390/foods12050920

**Published:** 2023-02-22

**Authors:** Zheyuan Hu, Penghui Zhao, Aimei Liao, Long Pan, Jie Zhang, Yuqi Dong, Jihong Huang, Weiwei He, Xingqi Ou

**Affiliations:** 1 School of Biological Engineering, Henan University of Technology, Zhengzhou 450001, China; 2 School of Food and Pharmacy, Xuchang University, Xuchang 461000, China; 3 State Key Laboratory of Crop Stress Adaptation and Improvement, College of Agriculture, Henan University, Kaifeng 475004, China; 4 Key Laboratory of Micro-Nano Materials for Energy Storage and Conversion of Henan Province, Institute of Surface Micro and Nano Materials, College of Chemical and Materials Engineering, Xuchang University, Xuchang 461000, China; 5 College of Life Science and Technology, Henan Institute of Science and Technology, Xinxiang 453003, China

**Keywords:** FWG, depression, behavior, neurotransmitters, intestinal microbes

## Abstract

Depression is a chronic mental illness with devastating effects on a person’s physical and mental health. Studies have reported that food fermentation with probiotics can enrich the nutritional values of food and produce functional microorganisms that can alleviate depression and anxiety. Wheat germ is an inexpensive raw material that is rich in bioactive ingredients. For example, gamma-aminobutyric acid (GABA) is reported to have antidepressant effects. Several studies concluded that *Lactobacillus plantarum* is a GABA-producing bacteria and can alleviate depression. Herein, fermented wheat germs (FWGs) were used to treat stress-induced depression. FWG was prepared by fermenting wheat germs with *Lactobacillus plantarum*. The chronic unpredictable mild stress (CUMS) model was established in rats, and these rats were treated with FWG for four weeks to evaluate the effects of FWG in relieving depression. In addition, the study also analyzed the potential anti-depressive mechanism of FWG based on behavioral changes, physiological and biochemical index changes, and intestinal flora changes in depressed rats. The results demonstrated that FWG improved depression-like behaviors and increased neurotransmitter levels in the hippocampus of CUMS model rats. In addition, FWG effectively altered the gut microbiota structure and remodeled the gut microbiota in CUMS rats, restored neurotransmitter levels in depressed rats through the brain–gut axis, and restored amino acid metabolic functions. In conclusion, we suggest that FWG has antidepressant effects, and its potential mechanism may act by restoring the disordered brain–gut axis.

## 1. Introduction

Depression is a chronic mental illness that negatively affects a person’s physical and mental health. Recurrent episodes of depression reduce the life expectancy and quality of life of an individual and enhance suicidal tendencies [1]. The global estimate of people suffering from depression is more than 350 million [2] and is expected to increase due to the increased stress in life. Depression is anticipated to top the total global burden of disease by 2030 [3], thus prioritizing the development of a treatment for depression. Standard pharmacological regimens, such as antidepressants, have limited efficacy, significant adverse effects, and rapid resistance [4], which might be attributed to the hitherto unclear pathogenesis of depression. Recently, brain–gut axis disorders, caused by dysbiosis of gut flora, have emerged as the main pathogenesis pathway of depression. Restoration of the gut microbiota in depressed patients can alleviate their depressive symptoms [5,6]. Fermented probiotic foods are emerging as healthy therapeutics that are associated with restoring the gut microbiota in depressive patients. Gut symbionts are found in gut–brain signaling, immunological homeostasis, and hormone regulation, and are known to reduce stress- and depression-related symptoms by regulating brain function [7]. Likewise, probiotics modulate the interaction between the gut and brain, thereby managing the effects of depression [8]. According to several studies, probiotic fermentation of raw materials can result in enriched nutritious products. These products can facilitate the proliferation of good gut bacteria and bioactive ingredients that can reduce depression and anxiety [9].

Wheat germs are a rich and inexpensive source of bioactive ingredients, containing high-quality proteins, amino acids, fats, vitamins, minerals, and other components [10]. Moreover, γ-aminobutyric acid (GABA), also found in wheat germs, has reported antidepressive effects [11]. The findings of several studies revealed that *Lactobacillus plantarum* is a GABA-producing bacteria that can alleviate depression [12,13]. Fermented wheat germs (FWGs) are obtained from the fermentation of wheat germs by *Lactobacillus plantarum* and comprise probiotics, prebiotics, protein, short-chain fatty acids, and GABA. Although there are a number of studies on probiotics for depression relief, there are still fewer studies on probiotic fermented foods for depression relief, especially *Lactobacillus plantarum*-FWG for depression relief, which has not been demonstrated by any other method before. In addition, most of the drugs developed for depression relief are based on the monoamine hypothesis using chemical methods, which usually have some side-effects on human health. In contrast, the study of *Lactobacillus plantarum*-FWG for depression alleviation adopts in a novel way a popular probiotic fermented food health therapy, in recent years, to develop new antidepressant functional foods that are healthy, green, and safe for the human body, providing a new direction for clinical treatment of depression. Finally, this study can fill the gap of fermented cereal products in the field of depression alleviation and provide new ideas for the development of cereal foods in the field of nutritional foods and functional foods.

**Figure 1:**
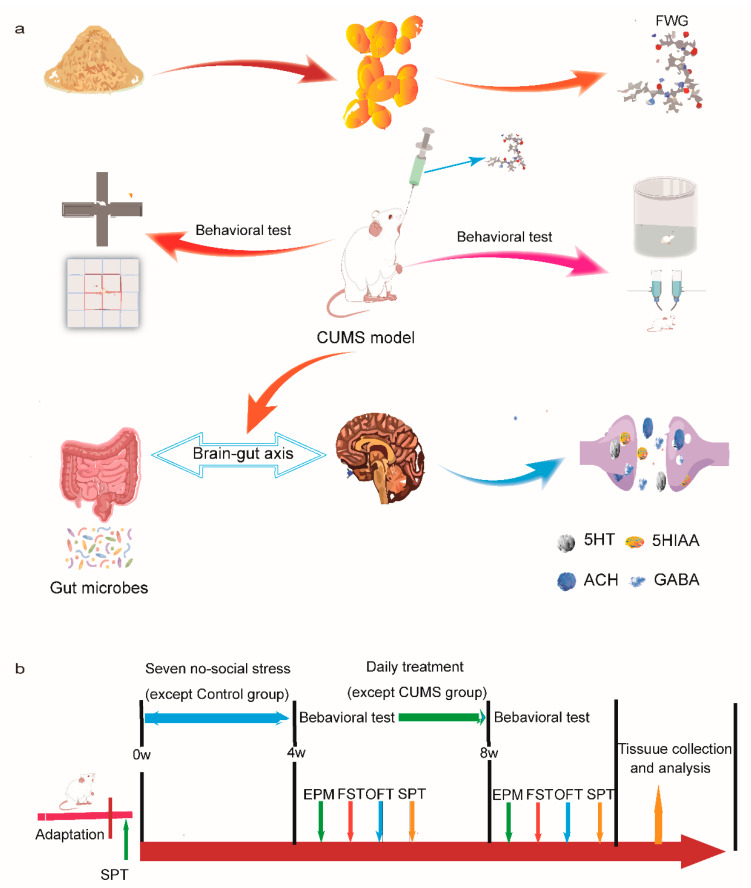
Experimental program overall design diagram (**a**) Schematic illustration for rational design of preparation of FWG for treatment of depression in rat by modulating gut microbes (Drawn by Figdraw). (**b**) Schematic diagram of a phased protocol for animal experiments. (FWG, Fermented wheat germs; CUMS, chronic unpredictable mild stress; 5-HT, 5-hydroxytryptamine; 5-HIAA, 5-hydroxyindoleacetic acid; ACH, acetylcholine; GABA, γ-aminobutyric acid; SPT, sucrose preference test; EMP, elevated plus maze experiment; FST, Forced swimming experiment; OFT, open field test.).

Herein, this study investigated the anti-depressive effects and potential mechanisms of *Lactobacillus plantarum*-FWG in depressed rats (Figure 1a). With a CUMS rat model, the animal behaviors, neurotransmitter index, and gut microbes were evaluated to verify the therapeutic efficacy of FWG to alleviate depression.

## 2. Materials and Methods

### 2.1. Materials

*Lactobacillus plantarum* (*M618*) was preserved in our laboratory. Bainong 207 defatted wheat germ powder was purchased from Henan Kunhua Biotechnology Co. (Anyang, China). Date palm GABA-positive compound tablets were obtained from MCOK BIOTECHNOLOGY (USA). The elevated plus maze and GABA content kit (plant source) were supplied by Zhengzhou Yixinyuan Instruments (Zhengzhou, China). 5-hydroxytryptamine (5-HT), 5-hydroxyindoleacetic acid (5-HIAA), and acetylcholine (ACH) assay kits were obtained from Equipment Co. Elabscience Biotechnology Co. GABA assay kits were supplied from Wuhan Fine Biotech Co. (Wuhan, China). The ZQZY-98 oscillating incubator was purchased from Shanghai Zhichu Instruments Co. (Shanghai, China). The digital display PHS-3C laboratory PH meter was purchased from Hefei Zhuoer Instrument Co. (Hefei, China). The XFH-40MA stainless-steel autoclave was purchased from Zhejiang Xinfeng Medical Instrument Co. (Shaoxing, China). The YSW-CB-V vertical purification bench was purchased from Shenzhen Yongshengwang Industrial Co., Ltd. (Shenzhen, China). The TG16-WS desktop high-speed centrifuge was purchased from Changsha Xiangyi Centrifuge Instrument Co. (Changsha, China). The FDU-21 freeze dryer was purchased from Tokyo RIKEN, Wakō, Japan. The SynergyHTX multifunctional enzyme labeler was purchased from BioTek, Winooski, VT, USA.

### 2.2. Preparation of FWG

The *Lactobacillus plantarum* was cultured in De Man Rogosa Sharpe(MRS) liquid media at 37 °C for 24 h to obtain a seed solution. The number of active bacteria in the seed solution of *Lactobacillus plantarum* was determined to be about 2 × 10^8^ CFU/mL by the dilution coating plate method.

After drawing on Wu’s study [14] as well as the results of single-factor (Lactobacillus plantarum inoculum, fermentation time, ratio of material, pH) experiments and response surface experiments in the pre-laboratory, FWG preparation conditions were determined with the goal of optimizing the GABA content in FWG. Wheat germ powder and 50 mmol/L of acetic acid-sodium acetate buffer salt were mixed in a ratio of 1:7, shaken well, and adjusted to pH 4.45 with NaOH/HCl. They were then sterilized using an autoclave at 105 °C for 30 min and cooled to room temperature, and 3.82% of the weight of wheat germ powder was inoculated with *Lactobacillus plantarum* seed solution (mL/g) in the ultra-clean table and mixed evenly. This was followed by fermentation in a shaker (37 °C, 100 rpm/min) for 24 h. After fermentation, the fermentation broth was centrifuged at 5000 r/min for 15 min to obtain FWG.

The FWG was stored at 4 °C and subsequently poured into several culture media plates (FWG, about 1/3 of each plate). Each plate was covered with cling film and dense and small holes were made on the surface of the cling film with a toothpick; then, the plates were placed in a refrigerator at −80 °C overnight to ensure that the FWG had been frozen into a solid form. Finally, the FWG was lyophilized according to the freeze dryer procedure. The lyophilized powder was analyzed for GABA content, using a GABA content kit. The results are displayed in Table 1. Excess lyophilized powder was subsequently stored in a −80 °C refrigerator.

### 2.3. Animals

Male SD rats weighing 170–190 g (SCXK(Yu)2019-0002) were obtained from the Henan Huaxing Experimental Animal Farm in China. Rats were kept in a room with a humidity of 40–60% and a temperature of 22–26 °C with water and meals readily available [15]. The animals were treated according to the National Institutes of Health (NIH) Guide for the Care and Use of Laboratory Animals. The Henan University of Technology ethics committee permitted the animal experiments conducted in this study.

### 2.4. CUMS Procedure and Treatment

Six groups were formed with seven male SD rats in each: blank control (no stress), depression model (CUMS), positive control (GABA), low-dose FWG, medium-dose FWG, and high-dose FWG. A week before the experiments, the rats were acclimatized and fed with 1% sucrose water. Following acclimatization feeding, the rats were weighed and evaluated for 1% sucrose preference. The rats were then subjected to seven stressors over four weeks, except for the blank control group. The stressors included day and night reversal, inclined cage at 45° (24 h), water fasting (24 h), wet bedding (400 mL of water spread onto the bedding for 10 h), tail clamping (1 min), swimming (4 °C water for 5 min), and strobing (300 times/min for 12 h) [16,17]. The animals were randomly given a stressor per day, and the same stressor was not delivered for three successive days. Their body weights were measured weekly. The elevated plus maze experiment (EPM), forced swimming test (FST), open field test (OFT), and sugar preference test (SPT) were conducted four weeks after concluding the modeling phase to examine the anxiety-depression-like behavior of the rats. The behavioral findings of the remaining groups were compared to the blank control group to determine the efficiency of the model. If the model responded poorly, the modeling period was further extended in two-week intervals (with a maximum of two months) until the optimum model response.

The CUMS group continued with the stressors for another four weeks, while the other groups stopped all stress stimulation. The following doses were used for gavage treatment intervention over four weeks: blank control group (normal saline 7 mL/kg·d), depression model group (normal saline 7 mL/kg·d), positive control group (26 mg/kg·d), low-dose FWG group (43 mg GABA/kg·d), medium-dose FWG group (43 mg GABA/kg·d), and high-dose FWG group (60 mg GABA/kg·d). The above experimental procedures are illustrated in Figure 1b.

### 2.5. Elevated plus Maze Experiment

EPM is an unconditioned reflex model that uses the animal’s exploratory nature in a different environment and the fear of an elevated open arm to create a conflicting state for the anxiolytic assessment of drugs. The rat was positioned in the middle of the maze with its head facing the open arm. Each experimental animal was placed in the same position thereafter, while the camera monitor was turned on to record the following indexes within five minutes: frequency of entries in the open arm (OE) and the closed arm (CE). The frequency with which the rat’s two forepaws fully enter into the corresponding arm was the criterion for determining the OE and CE frequencies [18]. Open-arm dwell time (OT) and closed-arm dwell time (CT) were measured in seconds. The feces of the rats were removed after each test and the maze was cleaned with 75% ethanol to mask the smell. The first and second elevated plus maze experiments were performed after the modeling and treatment interventions, respectively.

### 2.6. Forced Swimming Experiment

The behavioral test used in this experiment was developed by Porsolt et al. in 1977 for the evaluation of behavioral despair [19]. The experiment was split into two parts. The first part was pre-swimming, in which rats were confined in a transparent cylindrical container (water depth of 25–30 cm, water temperature 23 ± 1 °C) to swim for 15 min. The rats were then blown dry after pre-swimming and returned to their original cages. Experimental water was changed after each experiment to prevent external effects on subsequent experiments. The rats were placed in the pool again for a 6 min “test swim” after 24 h from the pre-swimming test and monitored. The duration of the static state of rats (e.g., body slightly curled, only nostrils exposed to maintain breathing, forepaws stopped digging, and hind paws occasionally paddled) during the last four minutes was observed. The static state is an animal response to abandon the thought of fleeing after it is unable to escape, which depicts a desperate predicament and behavioral despair. The first and second forced swimming experiments were performed after the modeling and treatment interventions, respectively.

### 2.7. Open Field Test (OFT)

The OFT was performed to study the spontaneous activity and exploratory behavior of rats [20]. The rats were placed in a 1 m × 1 m × 0.4 m observation box with black walls and a white floor, and red and blue lines separating the bottom surface into 16 equal squares (i.e., four red squares in the center and 12 blue squares in the periphery). Rats were put in the middle of the observation box to evaluate their horizontal and vertical activity scores. Within five minutes, the center region residence time, the number of squares traversed, and the frequency of upright times were collected [21]. To avoid interference from previous experiments, the experimental apparatus was washed with 75% ethanol after each experiment. The first and second OFTs were conducted after modeling and treatment interventions.

### 2.8. Sugar Preference Test (SPT)

The rats were trained with 1% sucrose water during laboratory acclimatization: Two bottles of 1% sucrose water were given for 24 h and then switched to two bottles of water for 24 h for the rats to acclimate to the sucrose water intake [22]. Following a 12 h fast, the first SPTwas performed. The rats were fed in respective cages, and two water bottles of similar shape were placed in each cage, 1% sucrose water and pure water. The 24 h experiment was conducted and the bottle positions were switched every 12 h [23]. Following that, both bottles were removed and weighed to determine each rat’s rate of sucrose preference. Sugar water preference rate = [sucrose water consumed/(sucrose water consumed + pure water expended)] 100%. The second and third sugar preference experiments were performed according to the above procedures after four weeks of modeling and treatment intervention.

### 2.9. Animal Handling and Tissue Dissection

The rats were fasted for 24 h after the behavioral test and deeply anesthetized with ether. Blood was collected from the eyeballs, and the rats were executed by severing their heads [24]. The rats were dissected on ice, and their heart, liver, spleen, lungs, kidneys, and cecum contents were removed, as well as the intact brain tissue, from which the brain, cerebellum, and cortex were separated. The aforementioned organs and tissues were preserved in liquid nitrogen for fast freezing and then in a −80 degrees Celsius refrigerator until usage.

### 2.10. Detection of Neurotransmitters

The rat hippocampus was dissected, weighed, and placed in a sterilized homogenization tube by reviewing the data combined, performing ELISA, and adding pre-chilled PBS buffer (in 2 additions) and Phenylmethylsulfonyl fluoride (PMSF) protease inhibitors at a working concentration of 1 nm/mL at a ratio of 1:9. The homogenization tube was rotated for 1–2 min to produce a 10% tissue homogenate. The levels of 5-hydroxytryptamine, 5-hydroxyindoleacetic acid, acetylcholine, and GABA in the hippocampus tissue homogenate supernatant were measured by the double antibody sandwich method [25].

### 2.11. 16S rRNA Sequence Analysis of the Gut Microbiota

To obtain microbial genomic DNA from samples of rat cecum contents, we employed the E.Z.N.A.^®^ Soil DNA Kit (Omega Bio-Tek, Norcross, GA, USA). A 1% agarose gel was utilized to investigate DNA extracts, and a NanoDrop 2000 UV-Vis spectrophotometer (Thermo Scientific, Wilmington, DE, USA) was used for measuring DNA concentration and purity. The thermal cycle PCR machine (Gene Amp 9700, ABI, USA) amplified the highly variable V3-V4 regions of the bacterial 16S rRNA gene 27 times with primers 338F (5′-ACTCCTACGGGAGGCAG-3′) and 806R (5′-GGACTACHVGGGTWTCTAAT-3′). The PCR reaction system was composed of 4 μL of 5× TransStart FastPfu buffer,10 ng of DNA template, 2 μL of 2.5 mM dNTPs, 0.8 μL of each upper and lower primer (5 μM), 0.4 μL of TransStart FastPfu DNA polymerase, and topped up to 20 μL with H_2_O. PCR reaction was carried out three times. The final PCR products were purified, quantified, and sequenced using Illumina’s MiseqPE300 platform. For microbiome analysis, raw sequences were quality-controlled using fastp (version 0.20.0) and spliced using FLASH (version 1.2.7) [26,27]. Chimeras were removed using UPARSE (version 7.1) [28]. Operable taxonomic units (OTUs) were constructed, and each sequence was classified and annotated using the RDP classifier (version 2.2), with a 70% comparison threshold set against the SILVA 16S rRNA database (version 138) [29]. The richness and diversity of samples were assessed using diversity analysis, and the results were subjected to the Wilcoxon rank sum test between the exponential groups. The principal coordinate analysis (PCoA) distance visualization approach was employed to investigate β-diversity in the microbial community. To investigate the variations in species composition across groups, colony bar plots at the family level and Wilcoxon rank-sum tests of substantially different colonies were utilized. PICRUSt2 predicted the effect of FWG on fecal microbiota function in CUMS rats.

### 2.12. Statistical Analysis

Experimental data on body weight, behavioral assessments, and neurotransmitter levels within the hippocampus were presented as mean ± standard error (SEM) of at least three independent experimental data samples. One-way ANOVA was used for statistical analysis. GraphPad Prism version 8.0 was used to run one-way ANOVA between groups (GraphPad software, Inc., La Jolla, CA, USA). In addition, ANOSIM analysis was performed using QIIME scripts. The Wilcoxon rank sum test was based on R’s stats package (version 3.3.1) and the Python scipy module.

## 3. Results

### 3.1. GABA Content in FWG Lyophilized Powder

In order to ensure that the GABA content in FWG lyophilized powder would not exceed the detection range of the kit when using the kit, different masses of FWG lyophilized powder were dissolved in 1 mL of water (mg/mL) for the determination, and the results in Table 1 showed that 10 mg of FWG lyophilized powder dissolved in 1 mL of water gave the best detection results, and the GABA content in FWG lyophilized powder was 19,797.54 ± 2719.322 (μg/g).

### 3.2. Amelioration of CUMS-Induced Depression-like Behavior in Rats by FWG

**Figure 2:**
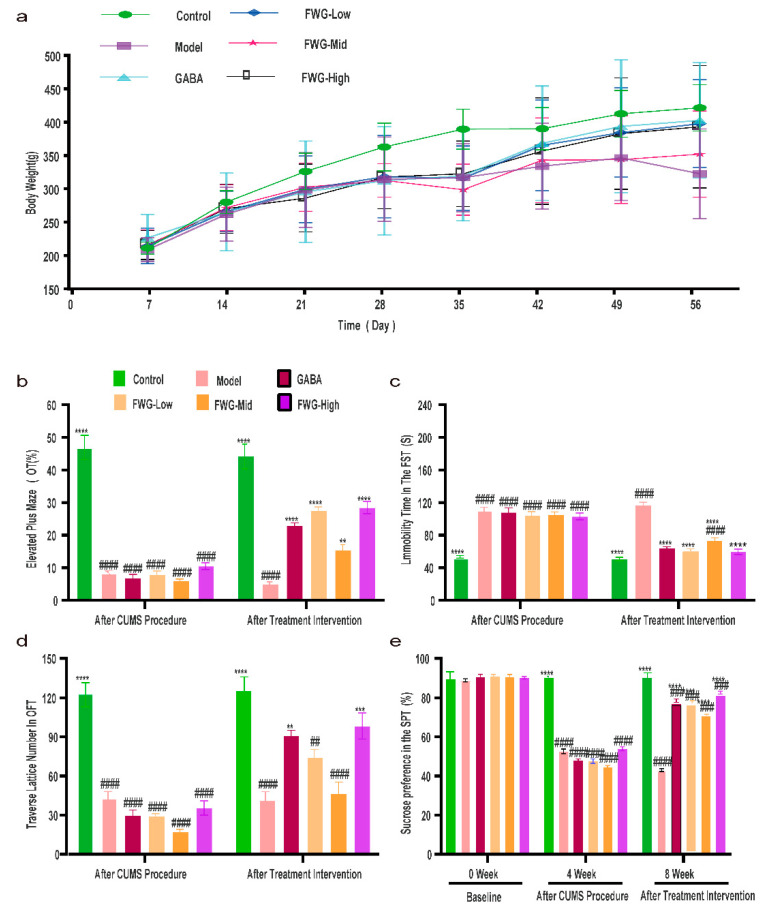
Effects of fermented wheat germ on the behavior of depressed rats. (**a**) Effect of FWG on body weight in CUMS rats. (**b**) Time to open arm as a percentage of total time (OT%). (**c**) Effect of fermented wheat germ on the immobility time of depressed rats in FST. (**d**) Number of lattices traversed by rats in OFT. (**e**) Effect of fermented wheat germ on sucrose preference in SPT. (** *p* < 0.01, *** *p* < 0.001, and **** *p* < 0.0001 indicate an appreciable difference compared to the model group. ## *p* < 0.01, ### *p* < 0.001, and #### *p* < 0.0001 represent that the difference was appreciable in contrast with the normal control group).

The influence of FWG on the weight of CUMS rats is depicted in Figure 2a. Before the test, there was no appreciable difference in the weight of rats in all groups. Rats stimulated by CUMS exhibited considerably lower body mass indexes than the control group after four weeks of CUMS modeling. The body weight indices of CUMS rats improved dramatically after four weeks of FWG administration. There was no significant difference between the high-dose FWG group and the blank control group.

The anxiolytic effect of drugs is usually assessed in the elevated plus maze experiment by OE and OT metrics, which are negatively correlated with anxiety. As demonstrated in Figure 2b, the OE of rats in the CUMS group was noticeably lower when compared to the control group (**** *p* < 0.0001). On the contrary, four weeks of treatment intervention restored the OE in the low-, medium-, and high-dose FWG groups to the level of the control group (N.S, # *p* > 0.05). Among these, the difference in the OE and OT of the high-dose FWG group was the largest as compared to the control group (**** *p* < 0.0001), thus indicating that FWG could improve the anxiety of depressed rats.

FST was designed to assess the level of despair in CUMS rats in a static state, and the study revealed that CUMS rats had a longer swimming immobility time and more pronounced despair [30]. As depicted in Figure 2c, CUMS rats had significantly more immobility time as compared to healthy controls (#### *p* < 0.0001). Following four weeks of intervention, the immobility time of rats in the low- and high-dose FWG groups decreased (**** *p* < 0.0001) and returned to a normal level (N.S), thus indicating that the high-dose FWG could improve the despair in depressed rats.

The number of traversal squares indicated the motor ability and exploratory behavior of rats, and the depressed rats have a lower number of traversed lattices. According to Figure 2d, the number of traversing lattices was appreciably lesser in CUMS rats than in the blank control group (#### *p*< 0.0001). After four weeks of FWG treatment, the low-, medium-, and high-dose FWG groups reported an increase in the frequency of traversing lattices in comparison to the CUMS model group. However, only the high-dose FWG group reported statistical significance (*** *p* < 0.001). Thus, a high-dose FWG treatment could improve the motor activity and independent exploratory behavior of depressed rats.

A lack of pleasure is a critical depressive trait that is assessed mainly by the percentage of sugar water ingested in the SPT [31]. In Figure 2e, the percentage of sugar water intake in each group of rats was not significantly different (N.S, *p* > 0.05). CUMS rats had a significantly lower percentage of sugar water intake than the blank controls (#### *p* < 0.0001). After four weeks of treatment intervention, rats in the low-, medium-, and high-dose FWG groups reported a significantly higher percentage of sugar water consumption than the CUMS model group (**** *p* < 0.0001, ## *p* < 0.001), indicating that FWG could improve the pleasure deficit in CUMS rats, but not fully restore them to normal levels.

### 3.3. Modulation of Neurotransmitters in the Hippocampus of CUMS Rats by FWG

**Figure 3:**
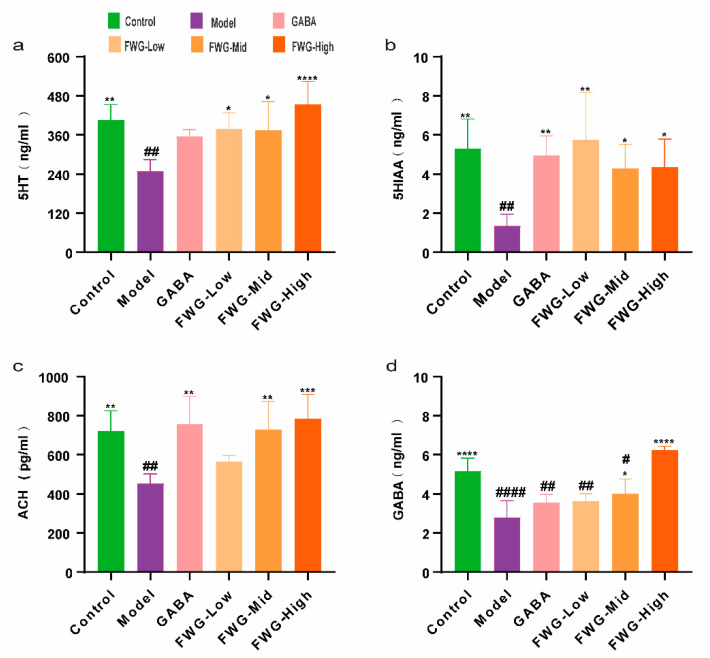
Effect of fermented wheat germ on neurotransmitters in the hippocampus of depressed rats. (**a**) Hippocampus 5-HT concentration. (**b**) Hippocampus 5-HIAA concentration. (**c**) Hippocampus ACH concentration. (**d**) Hippocampus GABA concentration. (* *p* < 0.05, ** *p* < 0.01, *** *p* < 0.001, and **** *p* < 0.0001 indicate an appreciable difference compared to the model group. # *p* < 0.05, ## *p* < 0.01, and #### *p* < 0.0001 represent that the difference was appreciable in contrast with the normal control group).

The levels of 5-HT, 5-HIAA, Ach, and GABA in the hippocampus of CUMS rats were significantly reduced in comparison with the normal control (## *p* < 0.01) (Figure 3). The low-dose FWG group exhibited a substantial increase in 5-HT and 5-HIAA levels after four weeks of treatment intervention as compared with the CUMS model group (* *p* < 0.05), which returned to normal levels that were comparable to the blank control group (N.S, # *p* > 0.05). The recovery of the other two neurotransmitters displayed an upward trend, but it was not significantly different. The medium-dose FWG group reported a significant increase in the levels of 5-HT, 5-HIAA, Ach, and GABA as compared with the CUMS model group (* *p* < 0.05, ** *p* < 0.01). The concentrations of 5-HT, 5-HIAA, and Ach were all restored to levels comparable to the control group (N.S, # *p* > 0.05), while GABA did not return to the normal level (# *p* < 0.05). In comparison to the model set, the high-dose FWG group exhibited slightly higher levels of 5-HT,5-HIAA, Ach, and GABA than the blank control group (* *p* < 0.05, **** *p* < 0.0001, # *p* > 0.05).

Figure 2 and Figure 3 demonstrate that the CUMS model was successfully established, and FWG had a similar impact on depression-like behaviors and neurotransmitter levels in the hippocampus of depressed rats. Furthermore, different doses of FWG had different degrees of improvement in depression, and the combined behavioral improvement results and neurotransmitter restoration effects highlighted that a high dose of FWG exhibited the strongest effect.

### 3.4. Analysis of Gut Microbe Diversity

**Figure 4:**
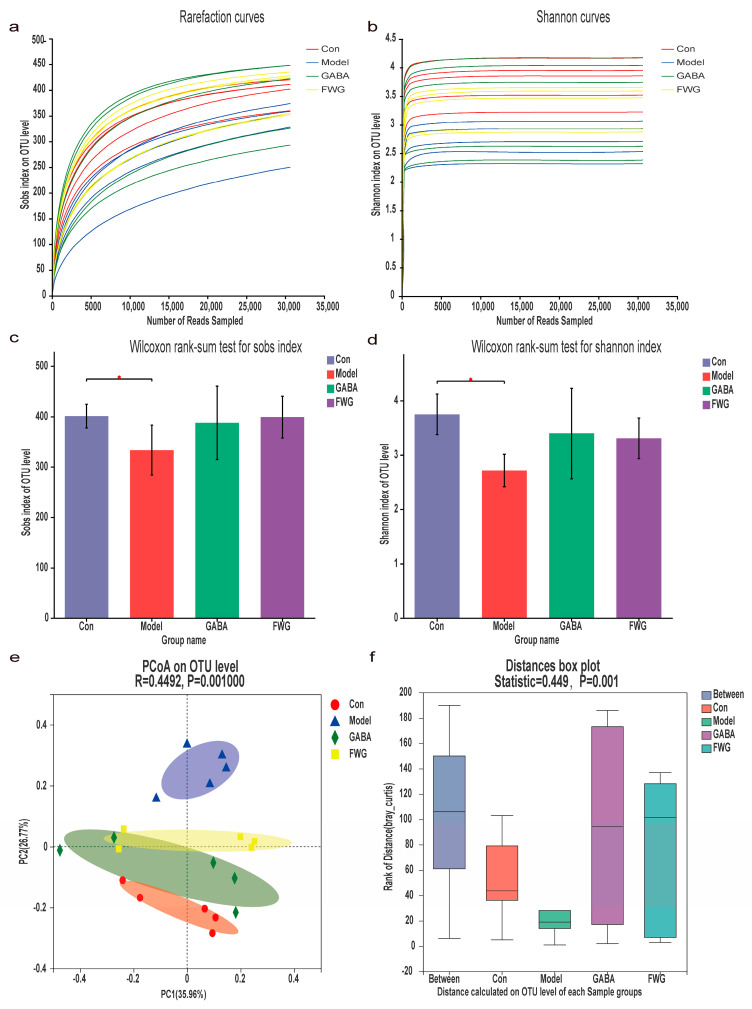
Analysis of gut microbe diversity. (**a**) Sample dilution curve based on Sobs index. (**b**) Sample dilution curve based on Shannon index. (**c**) Wilcoxon rank–sum test for Sobs index of differences between index groups. (**d**) Wilcoxon rank–sum test for Shannon index of differences between index groups. (**e**) PCoA analysis based on beta diversity analysis. (**f**) The results of an analysis of similarity (ANOSIM) based on PCoA. (* *p* < 0.05 indicates a statistically significant difference.)

The dilution curves in Figure 4a,b reached a plateau, indicating that the sequencing depth and coverage of the samples could be further analyzed. The α-diversity is reflected by community richness (Sobs index), while the species diversity of the samples is reflected by the community diversity index (Shannon index). There was a significant decrease in both species richness and diversity in the CUMS model group as compared with the blank control group *(p* < 0.05) (Figure 4c,d), and both species richness and diversity increased after treatment intervention with GABA and FWG *(p* > 0.05).

To differentiate samples with different microbial communities, we employed PCoA analysis based on β-diversity analysis. The rats in the CUMS model group had significantly different fecal microbiota than rats in the control group, and the fecal microbiotas of the GABA and FWG groups were similar to that of the blank control (Figure 4e). The results of an analysis of similarity (ANOSIM), based on PCoA scores, indicated a statistically remarkable divergence between all four groups (Figure 4f, R = 0.4492, *p* = 0.001).

### 3.5. Analysis of Intestinal Flora Composition at Different Classification Levels

**Figure 5:**
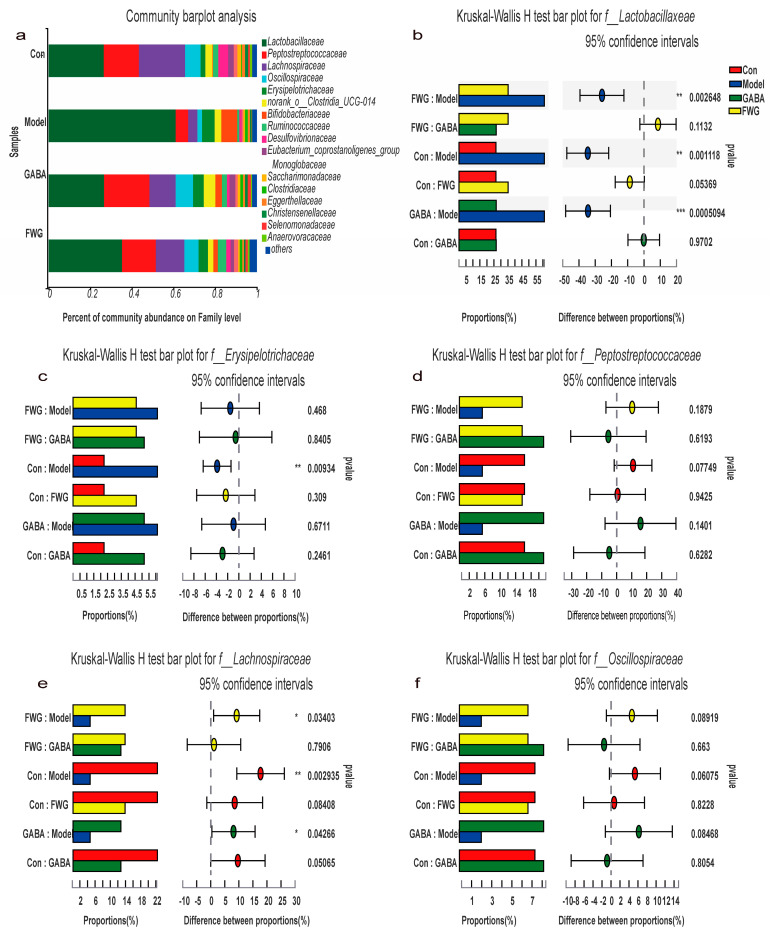
Differences in the species composition of gut microorganisms at the family level in different groups of samples. (**a**) Community bar–pot analysis at the family level. (**b**) Wilcoxon rank–sum test post hoc plots for *Lactobacillaceae*. (**c**) Wilcoxon rank–sum test post hoc plots for *Erysipelotrichaceae*. (**d**) Wilcoxon rank–sum test post hoc plots for *Peptostreptococcaceae*. (**e**) Wilcoxon rank–sum test post hoc plots for *Lachnospiraceae*. (**f**) Wilcoxon rank–sum test post hoc plots for *Oscillospiraceae*. (* *p* < 0.05, ** *p* < 0.01, *** *p* < 0.001 indicates a statistically significant difference.)

Differences in the species composition of gut microorganisms at the family level in different groups of samples are depicted in Figure 5. The model group had a higher abundance of both *Lactobacillaceae* and *Erysipelotrichaceae* and a lower abundance of *Peptostreptococcaceae*, *Lachnospiraceae*, and *Oscillospiraceae* than the other groups (Figure 5a). To determine the significance of their differences in different groups, the aforementioned families were treated to a multigroup rank sum test analysis. Figure 5b,c indicate that the abundance of *Lactobacillaceae* and *Erysipelotrichaceae* in the model set was considerably greater than in the blank control group *(p* < 0.01). After the treatment with GABA or FWG, the abundance of *Lactobacillaceae* decreased significantly until the level reached that of the blank control group *(p* > 0.05), while the abundance of *Erysipelotrichaceae* only decreased slightly. The abundance of *Peptostreptococcaceae* was lower in the model group in comparison to the blank control group *(p* > 0.05). Likewise, treatment with GABA or FWG significantly increased the abundance of *Peptostreptococcaceae* until the level reached that of the blank control group *(p* > 0.05) (Figure 5d). Meanwhile, the abundance of *Lachnospiraceae* was lower in the model group than in the blank control group *(p* < 0.01). After treatment with GABA or WFG, the abundance of *Lachnospiraceae* increased significantly until the level reached that of the blank control group (*p* > 0.05) (Figure 5e). The abundance of *Oscillospiraceae* was significantly lower in the model group relative to the control group, but the difference was not statistically significant (*p* > 0.05). The abundance of *Oscillospiraceae* increased significantly after GABA or FWG treatment until the level reached that of the blank control group (*p* > 0.05) (Figure 5f).

**Figure 6:**
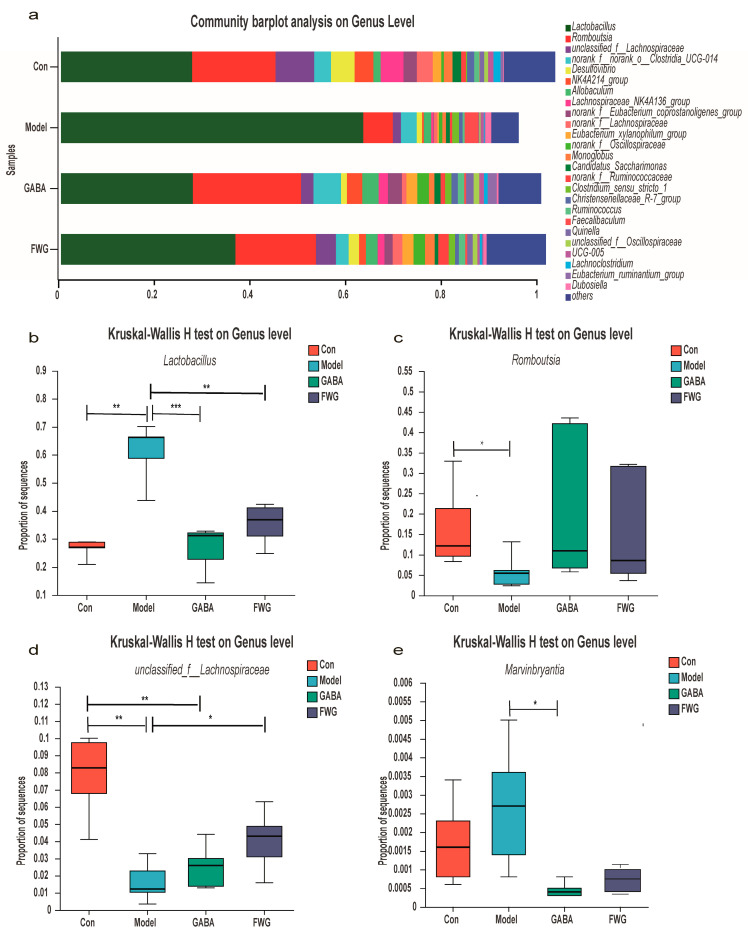
FWG regulated the composition of the gut microbiota at the genus level. (**a**) Community bar-pot analysis at the genus level. (**b**) Kruskal–Wallis H test box line graph for *Lactobacillus.* (**c**) Kruskal–Wallis H test box line graph for *Romboutsia*. (**d**) Kruskal–Wallis H test box line graph for *unclassified_f__Lachnospiraceae*. (**e**) Kruskal–Wallis H test box line graph for *Marvinbryantia*. (* *p* < 0.05, ** *p* < 0.01, *** *p* < 0.001 indicates a statistically significant difference.)

Figure 6 demonstrates that FWG regulated the composition of the gut microbiota at the genus level. The abundance of *Lactobacillus* and *Marvinbryantia* in the CUMS model group was higher than those in all the other groups, while the abundance of *unclassified_f__Lachnospiraceae* and *Romboutsia* was lower than those in all the other groups (Figure 6a). The Kruskal–Wallis H test was performed on the above genera, and the boxplot results are displayed in Figure 6b–e. The abundance of *Lactobacillus* was significantly higher in the model group than in the blank control group (*p* < 0.01) (Figure 6b). After treatment with GABA or FWG, the abundance of *Lactobacillus* significantly decreased until the level of the blank control group (*p* > 0.05). Likewise, the abundance of *Romboutsia* and *unclassified_f__Lachnospiraceae* in the model group was significantly lower than that of the normal control (*p* < 0.05, *p* < 0.01) (Figure 6c,d). After treatment with the FWG intervention, the abundance of *Romboutsia* and *unclassified_f__Lachnospiraceae* returned to levels that were close to that of the blank control group. The CUMS model group reported an increased abundance of *Marvinbryantia* compared to the control group (*p* > 0.05) (Figure 6e). After treatment with FWG, its abundance decreased to levels similar to the blank control group (*p* > 0.05).

### 3.6. Correlation Analysis between Gut Microbiota and Neurotransmitters

**Figure 7:**
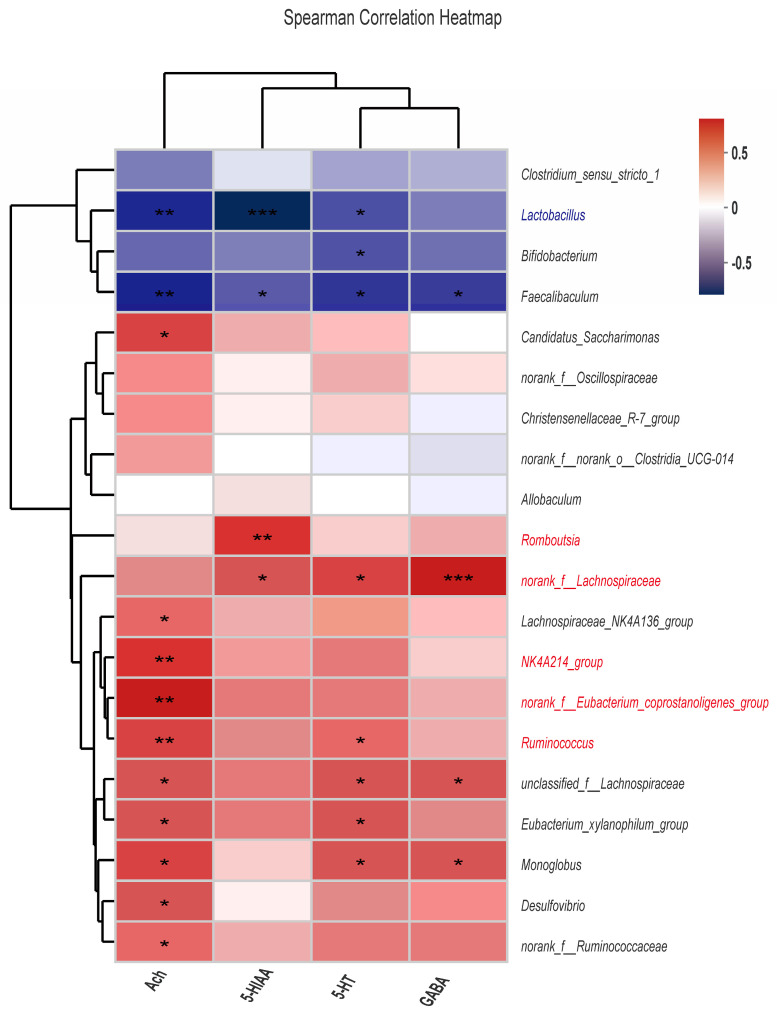
Correlation analysis between gut microbiota and neurotransmitters. (* *p* < 0.05, ** *p* < 0.01, *** *p* < 0.001 indicates a statistically significant difference.)

The analysis revealed 28 strong correlations between neurotransmitters and specific gut microflora (Figure 7). Among them, Ach was significantly and positively correlated with *Romboutsia*, *g__norank_f__Eubacterium_coprostanoligenes_group*, and *NK4A214_group*, while it was significantly and negatively correlated with *Lactobacillus*. Besides that, 5-HIAA was positively correlated with *Romboutsia* and negatively correlated with *Lactobacillus*. GABA and *norank_f__Lachnospiraceae* reported a significant positive correlation.

### 3.7. Impact of FWG on Gut Microbiota Function

**Figure 8:**
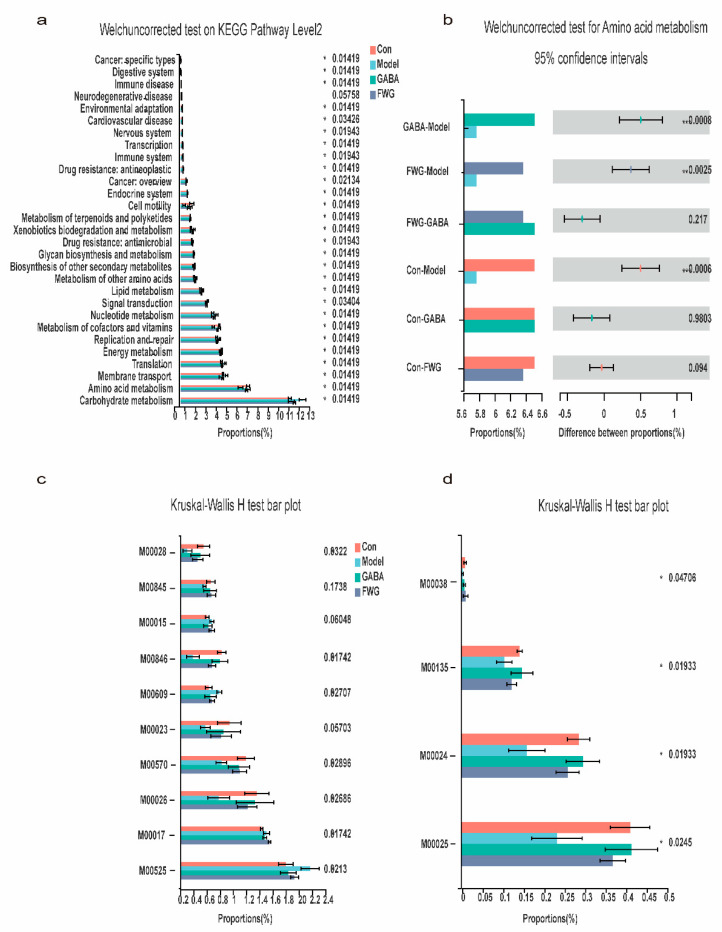
Effect of FWG on fecal microbiota function. (**a**) Differential pathways on KEGG Path Level2 in various groups of rats. (**b**) Wilcoxon rank −sum test of each group of rats in the amino acid metabolic pathway. (**c**,**d**) Differences between groups of rats related to the function of amino acid metabolism Module. (* *p* < 0.05, ** *p* < 0.01, *** *p* < 0.001 indicates a statistically significant difference.)

The analysis identified 29 KEGG pathways, with significant differences in KEGG Path Level 2 for each group of rats (Figure 8a). Among them, the carbohydrate and amino acid metabolic pathways were considered to be dominant, and depressed rats were usually characterized by a dysfunctional amino acid metabolism [32,33]. Hence, a rank sum test was performed on the amino acid metabolic pathways for each group of rats (Figure 8b). The results revealed that the CUMS model group had lower levels of amino acid metabolism than the normal group did (*p* < 0.01), implying dysfunctional amino acid metabolism. After treatment with GABA or FWG, the level of amino acid metabolism greatly improved and returned to levels near the blank control group (*p* > 0.05). The results of the KEGG Module for each group of rats were subjected to a rank sum test to further explore amino acid metabolism in depression (Figure 8c,d). The results demonstrated that there were 14 differential modules of amino acid metabolic functions among the groups of rats, including M00525, M00017, M00026, M00570, M00023, M00609, M00846, M00015, M00845, M00028, M00025, M00024, M00135, and M00038 (Table 2). The biosynthetic functions of lysine and proline were upregulated, while those of tryptophan, GABA, glutamate, isoleucine, and histidine were decreased in the CUMS model group. After GABA or FWG intervention, the effects were reversed.

## 4. Discussion

FWG improved the depression-like behaviors of CUMS rats in the current study, including improved weight loss, increased percentage of OT, decreased despair, improved mobility, and alleviated pleasure deficit symptoms. These findings are consistent with the results from previous studies, involving fermented American red ginseng (ARG) and fermented GABA oolong tea [34,35].

Neurotransmitter deficiency is the root cause of depression. Most antidepressants function by enhancing monoamine neurotransmitters in the brain [36,37]. However, these drugs have their limitations and side-effects in clinical practice. We evaluated the levels of 5-HT, 5-HIAA, Ach, and GABA in the hippocampus tissue to study the effect of FWG on neurotransmitter release. FWG was observed to increase the levels of 5-HT, 5-HIAA, Ach, and GABA in rat hippocampus tissues, which is consistent with earlier research [38]. Hence, FWG is generally recognized as safe (GRAS) and may be a potential alternative to treat depression-like behavior without side-effects.

According to emerging evidence, depression development may be linked to the gut flora [39,40,41]. Thus, we postulated that the anti-depressive effect of FWG might be correlated with intestinal microbiota and gut microbial function. The α-diversity data revealed that there was a remarkable decrease in both species’ richness and diversity in the CUMS model group in comparison with the blank control group (*p* < 0.05). Moreover, there was a general upward trend in both species’ richness and diversity after therapeutic intervention with GABA or FWG (*p* > 0.05). The PCoA results revealed that the intestinal flora of the CUMS model group differed significantly from that of the blank control group, validating the effects of chronic stress on the gut microbiota. The intestinal flora distribution in FGW rats was similar to that of the blank control group, suggesting that FWG intervention could change the structure of the intestinal flora. These findings were consistent with a recent study that discovered a noticeable change in the microbiota of depressed individuals after GABA-rich fermented milk intake [42]. Our findings are also congruent with previous research that found significant changes in the gut flora composition in rats with irritable bowel syndrome and depressed mice, which were fed with a barley and soybean fermentation mixture [43]. These results suggested that the anti-depressive effects of FWG might be mediated by the intestinal flora.

We observed that the abundance of *Lactobacillaceae* and *Erysipelotrichaceae* increased in the rat fecal microbiota of the CUMS model group. In addition, there was a lower abundance of *Peptostreptococcaceae*, *Lachnospiraceae*, and *Oscillospiraceae* in the CUMS model group than in the blank control group. There was also an increase in the abundance of *Lactobacillus* and *Marvinella* in the rat fecal microbiota of the CUMS model group, and a significant decrease in the abundance of *Romboutsia* and *unclassified _f__Lachnospiraceae* in the CUMS model group. However, the *Lactobacillus* strain might damage the host’s neurological function and lead to dysbiosis [44]. In some studies, the abundance of the highly immunogenic and inflammation-related *Erysipelotrichaceae* significantly increased in depressed patients, as well as those with IBS and neurodegenerative diseases [45,46]. Furthermore, there is a correlation between IBS and depression [47]. The study discovered that patients with depression had remarkably lower levels of *Peptostresptococcaceae* in their gut than healthy controls [48]. In a separate study, the abundance of *Peptostreptococcaceae* was lower in the CUMS model than in the control group [49]. From the above findings, we inferred that *Peptostreptococcaceae* is a group of depression-associated bacteria. On the contrary, the abundance of *Lachnospiraceae* can affect the metabolism of short-chain fatty acids, intestinal permeability, and neurotransmitter secretion [48], and its abundance was significantly reduced in CUMS rats [50]. The *unclassified _f__Lachnospiraceae* belonged to the *Lachnospiraceae* family, and a study on the intestinal flora of Chinese children with autism spectrum disorders (ASDs) reported that the abundance of *unclassified _f__Lachnospiraceae* was significantly reduced in ASD patients [51]. In summary, we concluded that *Lachnospiraceae* might be an important marker for identifying neurological diseases, especially depression. According to a study on the gut microbiota of depressed people, the abundance of *Oscillospiraceae* was reduced [52].

In a study on fish oil improving depression-like behavior and intestinal flora dysbiosis in chronically mildly stressed rats, CMS increased the abundance of *Marvinbryantia* [53]. In contrast, a reduced abundance of *Romboutsia* in the intestinal flora of patients had been reported in studies on neurological diseases, such as Alzheimer’s disease and neurodegenerative disorders [54,55].

It could be observed that FWG downregulated the abundance of Lactobacillaceae, Erysipelotrichaceae, Lactobacillus, and Marvinbryantia, while increasing the abundance of Peptostreptococcaceae, Lachnospiraceae, Oscillospiraceae, Romboutsia, and unclassified__f__Lachnospiraceae (Figure 5 and Figure 6). Taken together, our findings and the corroborating evidence from the literature uncovered a relationship between changes in the intestinal flora and depression-like symptoms, as well as the efficacy of FWG treatment in restoring the intestinal microbiota in CUMS rats.

Furthermore, the results of our Spearman correlation analysis (Figure 7) revealed an important role of gut microbiota in alleviating depression. A strong correlation between neurotransmitter levels and gut microbiota genus was also established, thus providing strong support for the brain–gut axis theory.

PICRUSt2 functional prediction data disclosed that the model group had dysfunctional amino acid metabolism as compared to the blank control group, with increased biosynthesis of lysine and proline and decreased biosynthesis of tryptophan, GABA, glutamate, isoleucine, and histidine. GABA or FWG intervention decreased the biosynthetic functions of lysine and proline and increased the biosynthetic functions of tryptophan, GABA, glutamate, and isoleucine. According to several studies, the genes related to lysine biosynthesis were significantly elevated in the fecal flora of depressed rats [56]. Hence, proline is significantly and positively correlated with the severity of depression. In addition, proline supplementation was reported to aggravate depression and microbial translocation in mice [57], as the accumulation of proline disrupted GABA production, glutamate release, and synaptic transmission. On the other hand, tryptophan affects the body’s mood, sleep quality, and appetite, and tryptophan levels were significantly reduced in depressed patients [58]. GABA, a naturally occurring non-protein amino acid, is an essential inhibitory neurotransmitter in the mammalian central nervous system. Studies have reported that GABA affects the monoamine levels in the brain, such that GABA deficiency triggers emotions such as anxiety, restlessness, fatigue, and depression [59]. Likewise, GABA supplementation alleviated sleep disturbance in mice [60]. Glutamate, an acidic amino acid, is a key excitatory neurotransmitter in the central nervous system, and its dysregulation might be a major cause of depression [61]. Low glutamate levels manifest as decreased excitability, resulting in a low mental state, depression, tiredness, and sleeplessness. Isoleucine aids in blood sugar regulation, and its deficiency can cause fatigue, melancholy, disorientation, and irritability. In summary, we speculated that FWG can alleviate depressive symptoms in CUMS rats by restoring the metabolic balance of lysine, proline, tryptophan, GABA, glutamate, and isoleucine.

It should be noted that the functional analysis of the intestinal flora in this study was performed by 16S rRNA sequencing technology to sequence and analyze very small DNA fragments in DNA extracts of the sample gut microbiota. Further mathematical computations were performed based on the sequencing data to predict the metabolic pathways. As a result, these data should be regarded as guidelines, and not as substantial evidence. With a rich database of whole-genome sequences of intestinal bacteria and a thorough annotation of their metabolic pathways, a precise understanding of intestinal microbial functions can be established. The involvement of lysine, proline, tryptophan, GABA, glutamate, and isoleucine in this study justified the requirement of future investigations, whereby untargeted metabolomics can be used to analyze more relevant metabolomics in cecum content samples.

Overall, our findings are consistent with previous research, indicating that CUMS resulted in gut microbiota dysregulation and that FWG could alleviate depressive symptoms. More research is required to fully elucidate the mechanisms of FWG in relieving depression and other neurological conditions.

## 5. Conclusions

In this study, we demonstrated that FWG improved depression-like behavior and increased neurotransmitter levels in the hippocampus of CUMS model rats. Our findings provided support for the role of intestinal microbiota in brain health and the anti-depressive effects of FWG in the gut-microbiota–brain axis. In particular, FWG could effectively change the intestinal microbiota structure of CUMS rats, remodel the intestinal microbiota, restore the neurotransmitter levels in depressed rats through the brain–gut axis, and recover amino acid metabolism functions. In conclusion, we believe that FWG has antidepressant potential. Our findings provide new ideas for the clinical treatment of depression and theoretical support for the development of safe novel antidepressant functional foods.

## Figures and Tables

**Table 1:** GABA content in FWG.

Mass of FWG Dissolved in 1 mL of Water (mg/mL)	Absorbance (Measuring Tube)	Absorbance (Control Tube)	GABA Content in FWG (μg/g)
1 mg	0.218	0.209	
1 mg	0.229	0.225	
1 mg	0.237	0.224	
10 mg	0.308	0.211	19,078.58
10 mg	0.328	0.239	17,509.94
10 mg	0.341	0.225	22,804.1
100 mg	0.967	0.187	
100 mg	0.869	0.212	
100 mg	0.947	0.175	

Notes: GABA content (μg/mL) = 1960.8 × (∆A + 0.0003), where ∆A = measuring tube absorbance − control tube absorbance. A value of ∆A hovering around 0 requires an increase in sample mass. If the absorbance of the measuring tube is greater than 0.6, the sample needs to be diluted.

**Table 2:** Annotation of Modules related to amino acid metabolism.

Modules Number	Module Description Information
M00525	Lysine biosynthesis, acetyl-DAP pathway
M00017	Methionine biosynthesis, aspartate ≥ homoserine
M00026	Histidine biosynthesis, PRPP ≥ histidine
M00570	Isoleucine biosynthesis, threonine ≥ 2-oxobutanoate
M00023	Tryptophan biosynthesis, chorismite ≥ tryptophan
M00609	Cysteine biosynthesis, methionine ≥ cysteine
M00846	Siroheme biosynthesis, glutamate ≥ siroheme
M00015	Proline biosynthesis, glutamate ≥ proline
M00845	Arginine biosynthesis, glutamate ≥ acetylcitrulline
M00028	Ornithine biosynthesis, glutamate ≥ ornithine
M00025	Tyrosine biosynthesis, chorismite ≥ tyrosine
M00024	Phenylalanine biosynthesis
M00135	GABA biosynthesis, eukaryotes, putrescine ≥ GABA
M00038	Tryptophan metabolism, tryptophan≥ kynurenine

## Data Availability

The datasets generated for this study are available on request to the corresponding author.
